# Pseudo-Hypobicarbonatemia in Patients With Hypertriglyceridemia

**DOI:** 10.7759/cureus.55787

**Published:** 2024-03-08

**Authors:** Cami J Good, Yazeed G Sweedan, Sanjana Kamat, Chudamani Giri

**Affiliations:** 1 Family Medicine, Conemaugh Memorial Medical Center, Johnstown, USA; 2 Internal Medicine, Conemaugh Memorial Medical Center, Johnstown, USA

**Keywords:** hepatosplenomegaly, acute pancreatitis, epigastric pain, hypertriglyceridemia (htg), hypobicarbonatemia, serum bicarbonate, anion-gap metabolic acidosis, diabetic ketoacidosis (dka)

## Abstract

Hypobicarbonatemia with an elevated anion gap on a metabolic panel is frequently the initial marker of a life-threatening condition such as diabetic ketoacidosis in a patient with epigastric pain. The two commonly used means of measuring bicarbonate levels are direct measurement from a metabolic panel and calculated measurement from arterial blood gas. In this case report, we would like to highlight a potentially serious deficiency in one of these two means and how it may lead to a dangerous misdiagnosis and subsequent mismanagement. We also shine a light on potential measures to counteract or prevent this undesirable outcome.

## Introduction

Hypobicarbonatemia with an elevated anion gap on a metabolic panel is frequently the initial marker of a life-threatening condition such as diabetic ketoacidosis in a patient with epigastric pain [1]. The two commonly used means of measuring bicarbonate levels are direct measurement from a metabolic panel and calculated measurement from arterial blood gas (ABG) [1]. It was postulated that the light-scattering effect of hyperlipidemia interfered with the enzymatic/spectro-photometric method, resulting in factitious low bicarbonate levels [2]. In this case report, we highlight a potentially serious deficiency in one of these two means and how it may lead to a dangerous misdiagnosis and subsequent mismanagement. In addition, we shine a light on potential measures to counteract or prevent this undesirable outcome.

## Case presentation

A 46-year-old female with a medical history significant for insulin-dependent diabetes mellitus, hypertriglyceridemia, and recurrent pancreatitis presented to the emergency department with a chief complaint of epigastric pain of one-week duration. Further questioning failed to unveil any additional symptoms or pertinent details. Physical examination was remarkable for moderate epigastric tenderness on deep palpation. Initial serum testing was significant for hyperglycemia with anion-gap metabolic acidosis (Table 1). Additional laboratory testing to more accurately diagnose the cause of the patient’s epigastric pain revealed normal troponin, lipase, aactic acid, and beta-hydroxybutyrate levels (Table 1). Hepatic functional panel as well as inflammatory markers including erythrocyte sedimentation rate and C-reactive protein were also found to be within normal limits. Urine testing showed marked glucosuria without ketones (Table 2). Microbiological testing including coronavirus disease 2019 polymerase chain reaction, rapid influenza A/B antigen, blood cultures, and urine cultures were all negative.

**Table 1:** Serum laboratory test results during the hospital stay. ED = emergency department

Serum	Reference range	Patient’s values (ED)	Patient’s values (day 2)	Patient’s values (day 3)
White blood cell count	3.1–8.5 10^3^/µL	5.54	4.95	4.51
Sodium	136–145 mmol/L	125	135	131
Potassium	3.5–5.1 mmol/L	4.0	4.4	4.7
Chloride	98–107 mmol/L	93	103	100
Bicarbonate	23–31 mEq/L	<10	20	21
Creatinine	0.55–1.30 mg/dL	1.10	0.8	0.8
Aspartate aminotransferase	5–34 U/L	27	-	-
Alanine aminotransferase	≤55 U/L	17	-	-
Alkaline phosphatase	40–150 U/L	95	-	-
Lactic acid	0.5–2.0 mmol/L	1.2	-	-
Erythrocyte sedimentation rate	0–20 mm/hour	18	-	-
C-reactive protein	0–0.8 mg/dL	0.5	-	-
Procalcitonin	0–0.05 ng/mL	<0.04	-	-
High-sensitivity troponin I	0–53 ng/L	<2.5	-	-
Hemoglobin A1c	≤6.0%	12.8	-	-
Blood glucose	70–105 mg/dL	457	163	122
Beta-hydroxybutyrate	0.02–0.27 mmol/L	0.12	-	-
Triglycerides	≤150 mg/dL	-	>1,000	>1,000
Lipase	12–53 U/L	47	37	-

**Table 2:** Urine laboratory test results during the hospital stay. ED = emergency department; HPF = high-power field

Urine	Reference range	Patient’s values (ED)
Urine glucose	0 mg/dL	≥1000
Specific gravity	1.005–1.030	1.044
Urine ketones	110–250 mmol/L	Negative
Protein	0 mg/dL	Negative
Red blood cells	1–2/HPF	0–2
White blood cells	0–5/HPF	11–20
Bacteria	0/HPF	1+
Hyaline casts	0/LPF	0–8

**Figure 1:**
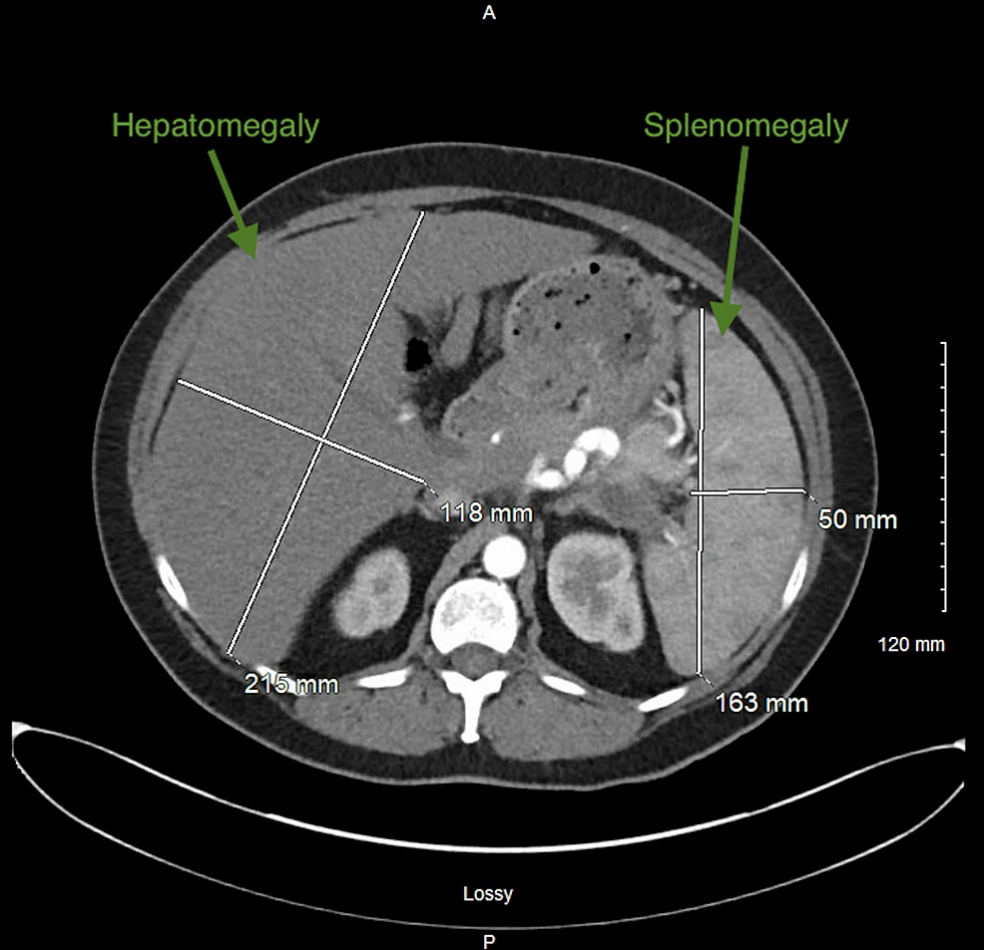
Enhanced computed tomography of the abdomen and pelvis. Liver dimensions: 11.8 x 21.5 x 24.3 cm. Spleen dimensions: 16.3 x 5.0 x 15.9 cm.

An enhanced computed tomography of the abdomen and pelvis revealed prominent atherosclerotic vascular calcifications of the abdominal aorta, along with hepatomegaly and splenomegaly (Figure 1).

In the emergency department, a preliminary diagnosis of diabetic ketoacidosis with suspicion of acute pancreatitis manning the role of the trigger was made. The hospital’s diabetic ketoacidosis protocol was released leading to the initiation of intravenous fluids and titratable insulin infusion before admitting the patient to the internal medicine service.

Upon review of the initial evaluation, the preliminary diagnosis of diabetic ketoacidosis was not accepted due to the inability to reasonably explain the absence of ketones in the serum and urine. The trigger “acute pancreatitis” was also rejected due to the normal lipase level and absence of relevant signs on the enhanced computed tomography. Fortunately, further testing revealed hypertriglyceridemia as the culprit responsible for the patient’s epigastric pain and pseudo-hypobicarbonatemia (Table 1). The patient’s insulin infusion was continued while her diet was modified to restrict both fats and carbohydrates. The epigastric pain severity as well as the serum bicarbonate level were utilized as a surrogate indicator of response to therapy. The patient experienced a gradual but dramatic improvement with complete resolution of symptoms and eventual discontinuation of the insulin infusion. Soon thereafter, she was discharged on a new dietary regimen, Icosapent-ethyl, fenofibrate, and a referral to the endocrinology clinic. Triglyceride level was not utilized to monitor response to therapy due to our hospital’s analyzer not quantifying values above 1,000 mg/dL. However, the serum triglyceride level was re-tested on the day of discharge and had dropped down to 449 mg/dL.

## Discussion

Hypobicarbonatemia with an elevated anion gap on a metabolic panel is frequently the initial marker of a life-threatening condition such as diabetic ketoacidosis in a patient with epigastric pain [1].

The two commonly used means of measuring bicarbonate levels are direct measurement from a metabolic panel and calculated measurement from ABG [1]. Direct measurement via a metabolic panel utilizes the total CO_2_ as an accurate surrogate for the bicarbonate level as it is 95% bicarbonate while dissolved CO_2_ and carbonic acid constitute the remaining ~5% [1]. For measuring total CO_2_, automated chemistry analyzers use either an indirect ion-selective electrode or the enzymatic/spectro-photometric method [1]. With the spectro-photometric method, certain wavelengths of light get absorbed based on the specific properties of the analyte [1]. The second method utilizes an ABG sample and the Henderson-Hasselbalch equation to calculate the bicarbonate level from the measured pH and partial pressure of CO_2_ [1].

Rifkin et al. described the first case of pseudo-hypobicarbonatemia caused by profound hyperlipidemia. It was postulated that the light-scattering effect of hyperlipidemia interfered with the enzymatic/spectro-photometric method, resulting in factitious low bicarbonate levels. This factious measurement of bicarbonate levels resolved after treatment with lipid-clearing agents [2]. Varghese et al. analyzed laboratory data of patients with triglycerides greater than 1,000 mg/dL on admission, serum bicarbonate level of less than 12 mEq/L, and had an ABG analysis done with six hours of venous sampling. The analysis detected spuriously low bicarbonate levels due to lipemic interference in 60% of the severe hypertriglyceridemia sample [3].

In summary, due to the reliance on photometric means to analyze metabolic panels, the light-scattering effect of conditions such as hypertriglyceridemia may result in falsely low bicarbonate readings and subsequent misdiagnosis [1]. The adverse effects of such misdiagnosis affect the immediate treatment of a patient, as well as their long-term management. An example of this can be illustrated by the incorrect discontinuation of empagliflozin, a drug of mortality benefit, due to the improper attribution of a falsely low bicarbonate level to euglycemic diabetic ketoacidosis.

## Conclusions

The light-scattering effect of conditions such as hypertriglyceridemia may result in falsely low bicarbonate readings and subsequent misdiagnosis when optometric analysis of metabolic panels is done. The adverse effects of such misdiagnosis can affect the immediate and long-term treatment of patients. To counteract and prevent this misdiagnosis, several measures have been proposed. Of these measures, we advocate for raising physician awareness of this laboratory artifact and promoting the use of ABG to confirm bicarbonate levels that are less than 12 mEq/L. Finally, we would like to emphasize the importance of utilizing ketone testing by serum beta-hydroxybutyrate or urine analysis to accurately detect euglycemic diabetic ketoacidosis.
